# Specific microRNA signatures in exosomes of triple-negative and HER2-positive breast cancer patients undergoing neoadjuvant therapy within the GeparSixto trial

**DOI:** 10.1186/s12916-018-1163-y

**Published:** 2018-10-10

**Authors:** Ines Stevic, Volkmar Müller, Karsten Weber, Peter A. Fasching, Thomas Karn, Frederic Marmé, Christian Schem, Elmar Stickeler, Carsten Denkert, Marion van Mackelenbergh, Christoph Salat, Andreas Schneeweiss, Klaus Pantel, Sibylle Loibl, Michael Untch, Heidi Schwarzenbach

**Affiliations:** 1 Department of Tumor Biology, University Medical Center Hamburg-Eppendorf, Martinistraße 52, 20246 Hamburg, Germany; 2 Department of Gynecology, University Medical Center Hamburg-Eppendorf, Hamburg, Germany; 3 GBG Forschungs GmbH, Neu-Isenburg, Germany; 4 Department of Gynecology and Obstetrics, University Hospital Erlangen, Comprehensive Cancer Center Erlangen-EMN, Friedrich-Alexander University Erlangen-Nuremberg, Erlangen, Germany; 5 University Women’s Hospital, Frankfurt, Germany; 6 Center for Gynecological Oncology at University Women’s Hospital, Heidelberg, Germany; 7 Mammazentrum Hamburg, Hamburg, Germany; 8 Universitätsklinikum Aachen, Aachen, Germany; 9 Charite Berlin, Institute of Pathology and German Cancer Consortium (DKTK), Partner Site, Berlin, Germany; 10 Universitätsklinikums Schleswig-Holstein Kiel, Kiel, Germany; 11 Hämatoonkologische Schwerpunktpraxis, Munich, Germany; 12 Universitätsklinikum Heidelberg, Heidelberg, Germany; 13 Helios Kliniken Berlin-Buch, Berlin, Germany

**Keywords:** MicroRNAs, Exosomes, Breast cancer, Triple negative, HER2-positive, Pathological complete response, Neoadjuvant therapy

## Abstract

**Background:**

The focus of this study is to identify particular microRNA (miRNA) signatures in exosomes derived from plasma of 435 human epidermal growth factor receptor 2 (HER2)-positive and triple-negative (TN) subtypes of breast cancer (BC).

**Methods:**

First, miRNA expression profiles were determined in exosomes derived from the plasma of 15 TNBC patients before neoadjuvant therapy using a quantitative TaqMan real-time PCR-based microRNA array card containing 384 different miRNAs. Forty-five miRNAs associated with different clinical parameters were then selected and mounted on microRNA array cards that served for the quantification of exosomal miRNAs in 435 BC patients before therapy and 20 healthy women. Confocal microscopy, Western blot, and ELISA were used for exosome characterization.

**Results:**

Quantification of 45 exosomal miRNAs showed that compared with healthy women, 10 miRNAs in the entire cohort of BC patients, 13 in the subgroup of 211 HER2-positive BC, and 17 in the subgroup of 224 TNBC were significantly deregulated. Plasma levels of 18 exosomal miRNAs differed between HER2-positive and TNBC subtypes, and 9 miRNAs of them also differed from healthy women. Exosomal miRNAs were significantly associated with the clinicopathological and risk factors. In uni- and multivariate models, miR-155 (*p* = 0.002, *p* = 0.003, respectively) and miR-301 (*p* = 0.002, *p* = 0.001, respectively) best predicted pathological complete response (pCR).

**Conclusion:**

Our findings show a network of deregulated exosomal miRNAs with specific expression patterns in exosomes of HER2-positive and TNBC patients that are also associated with clinicopathological parameters and pCR within each BC subtype.

**Electronic supplementary material:**

The online version of this article (10.1186/s12916-018-1163-y) contains supplementary material, which is available to authorized users.

## Background

Breast cancer (BC) comprises several subtypes that are in clinical routine defined by estrogen receptor (ER), progesterone receptor (PR), and human epidermal growth factor receptor 2 (HER2) status. Each BC subtype exhibits varied responses to different therapeutic regimens. Triple-negative (TNBC) and HER2-positive tumors are associated with a worse prognosis, a more aggressive clinical outcome, and a higher risk for relapse than luminal-like tumors that are positive for hormone receptors [1].

Since particular microRNA (miRNA) signatures are associated with BC subtypes and aggressiveness, as well as patient response to drug therapy and clinical outcome [2, 3], they open up new approaches for the development of non-invasive diagnostic and therapeutic tests. The deregulated expression of miRNAs in cancer may among others be caused by their frequent location in fragile chromosomal regions harboring DNA amplifications, deletions or translocations [4]. As evolutionary conserved family, these small non-coding RNA molecules inhibit post-transcriptionally gene expression by binding to complementary sequences in the 3′ untranslated-region (3′UTR) of their target mRNAs [5]. MiRNAs circulate highly stable in human blood [6]. They are released into the blood circulation either passively by apoptosis and necrosis or actively by exosomes from multiple cell types [7, 8]. The process of sorting and packaging of miRNAs into exosomes depends on the cell origin, and is selective, favoring certain miRNAs for exosomal cargo to others [9, 10]. Exosomes are small membrane vesicles in size of 30–100 nm [11]. They can mediate cell-to-cell communication by transferring proteins, lipids, and nucleic acids between donor and recipient cells, resulting possibly in modulation of the recipient cells. It is assumed that tumor-derived exosomes can transform normal, wild-type cells into malignant cells [12, 13]. In this manner, they stimulate cellular signaling and regulate metabolic functions and homeostasis of hematopoietic cells [14, 15]. MiRNAs derived from cancer-associated exosomes have been implicated in supporting or restraining tumor growth, conferring drug resistance, promoting recurrence, and preparing a metastatic niche [10]. Considering their biologic relevance, strategies to interfere with loading or delivery of exosomal oncogenic miRNAs might be used as a therapeutic approach.

Currently, TNBC is a focus of intense research since treatment options beyond chemotherapy are urgently required. In contrast, many options for HER2-positive patients exist but the optimal combination strategies are unclear, and clarifying the mechanisms of resistance is required. In both settings, it is important to improve the insights into the biology of tumor progression in the context of therapy. Neoadjuvant treatment strategies offer short-term results of treatment efficacy by evaluation of pathological complete response (pCR). Since this response is associated with long-term outcome, the treatment strategy is now used for the clinical evaluation of treatment strategies in BC patients.

In this study, we determined the expression of miRNA profiles in circulating exosomes of BC patients before neoadjuvant therapy within a randomized phase II neoadjuvant GeparSixto trial using quantitative TaqMan real-time PCR-based microRNA array cards. We detected a significant difference in the exosomal miRNA patterns between TNBC and HER2-positive patients. The packaging of particular miRNAs in exosomes was associated with clinicopathological and risk factors, and predicted pCR.

## Methods

### Study populations

**Fig. 1:**
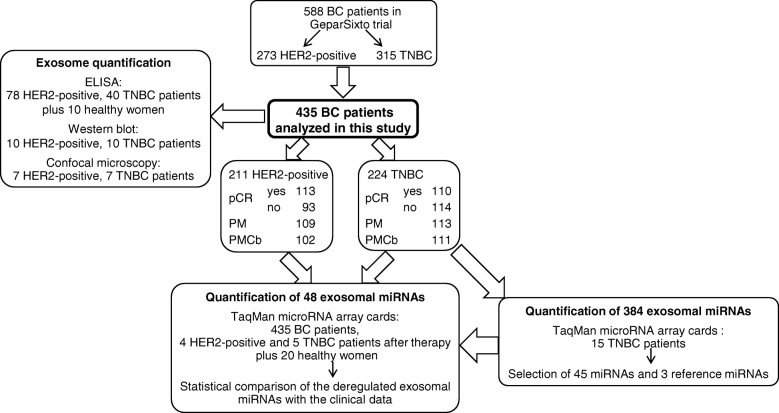
Workflow of the present study. pCR, pathological complete response; pM, non-carboplatin arm; pMCb, carboplatin arm

Within the multicenter GeparSixto trial from August 2011 to December 2012, BC patients were randomized to receive 18 weeks of neoadjuvant treatment with paclitaxel (80 mg/m^2^/week) and non-pegylated liposomal doxorubicin (20 mg/m^2^/week) with or without addition of carboplatin (AUC 2.0–1.5/week) [16]. Hormone-receptor status, HER2 status, and Ki67 expression were centrally confirmed prior to randomization. Plasma samples of 211 HER2-positive and 224 TNBC patients were collected directly before neoadjuvant therapy. After therapy, plasma samples of 4 HER2-positive and 5 TNBC patients were available. Median age of BC patients was 47 years and ranged from 21 to 78 years. Detailed patient characteristics are summarized in Table 1 (categorial variables) and Additional file 1: Table S1 (continuous variables). During 2016, plasma samples were collected from 20 healthy women with no history of cancer and in good health based on self-report (median age 55, range 47 to 69). Regarding blood processing, uniform management concerning the specific described protocols was performed. Blood collection and experiments were performed in compliance with the Helsinki Declaration and were approved by the ethics committee (Ethik-Kommission der Ärztekammer Hamburg, Hamburg). Plasma samples from 435 BC patients and 20 healthy women were analyzed with different techniques as described below, and sample flow is depicted in Fig. 1.

**Table 1:** Breast cancer patient characteristics (categorial variables)

Parameters		All BC patients analyzed in this study	HER2-positive patients	TNBC patients	All BC patients in GeparSixto trial	*p* value*
Total		435 (100.0%)	211 (100.0%)	244 (100.0%)	588 (100.0%)	
Subtype	HER2-positive patients	211 (48.5%)	Subgroups of all BC patients analyzed in this study		273 (46.4%)	0.0909
	TNBC patients	224 (51.5%)			315 (53.6%)	
Age	< 50	249 (57.2%)	120 (56.9%)	129 (57.6%)	341 (58.0%)	0.5684
	≥ 50	186 (42.8%)	91 (43.1%)	95 (42.4%)	247 (42.0%)	
Lymph node metastasis	N0	240 (56.1%)	106 (50.7%)	134 (61.2%)	338 (58.7%)	0.0333
	N+	188 (43.9%)	103 (49.3%)	85 (38.8%)	238 (41.3%)	
	Missing	7	2	5	12	
Tumor size	T1–2	365 (84.3%)	167 (79.9%)	198 (88.4%)	499 (85.2%)	0.3570
	T3–4	68 (15.7%)	42 (20.1%)	26 (11.6%)	87 (14.8%)	
	Missing	2	2	0	2	
Grading	G1–2	151 (34.7%)	93 (44.1%)	58 (25.9%)	207 (35.2%)	0.6944
	G3	284 (65.3%)	118 (55.9%)	166 (74.1%)	381 (64.8%)	
Lymphocyte predominant breast cancer	pos.	108 (25.1%)	44 (21.4%)	64 (28.6%)	142 (24.4%)	0.5825
	neg.	322 (74.9%)	162 (78.6%)	160 (71.4%)	439 (75.6%)	
	Missing	5	5	0	7	
Therapy arm	PM	222 (51.0%)	109 (51.7%)	113 (50.4%)	293 (49.8%)	0.3478
	PMCb	213 (49.0%)	102 (48.3%)	111 (49.6%)	295 (50.2%)	
Pathological complete response (pCR)	Yes	223 (51.3%)	113 (53.6%)	110 (49.1%)	296 (50.3%)	0.4540
	No	212 (48.7%)	98 (46.4%)	114 (50.9%)	292 (49.7%)	

*PM* non-carboplatin treatment arm, *PMCb* carboplatin treatment arm

*Characteristics of patients were compared between patients with analyzed samples and all patients of GeparSixto study (modified intend-to-treat population) using Fisher’s exact tests

### Verification of hemolysis in plasma samples

To avoid quantifying exosomal miRNAs in hemolytic plasma samples that may influence our results, we performed hemoglobin measurements by spectral analysis [17]. In 7 ml of whole blood, red blood cells were lysed by erythrocyte lysis buffer (containing 0.3 M sucrose, 10 mM Tris pH 7.5, 5 mM MgCl2 and 1% Triton X100). A dilution series (1:1, 1:3, 1:4, 1:6, 1:8, 1:10, 1:12, 1:14, 1:18, 1:20) of lysed red blood cells in plasma was prepared that served as a standard curve for the measurement of hemolysis in all plasma samples. Fifty microliters of each plasma sample (standard and plasma of interest) was measured in duplicates on a Microplate reader (Tecan, Männerdorf, Switzerland). Absorbance peaks at 414, 541, and 576 nm were indicative for free hemoglobin, with the highest peak at 414 nm. The higher the absorbance in samples is, the higher is the degree of hemolysis. The average values and standard deviations were calculated from the duplicates (see Additional file 1: Figure S1).

### Isolation of total exosomes from plasma

Total exosomes were isolated by ExoQuick (BioCat, Heidelberg, Germany) according to the manufacturer’s instructions. Briefly, 550 μl of plasma, removed from cells and debris by two centrifugation steps at 3000*g* for 15 min, was incubated with 120 μl ExoQuick exosome precipitation solution at 4 °C, for 30 min. Following centrifugation at 1500*g* for 30 min, the exosomes were precipitated and then resuspended in 50 μl PBS (phosphate-buffered saline) buffer (Life Technologies, Darmstadt, Germany).

### Visualization of exosomes using confocal microscopy

The isolated exosomes were labeled by the Exo-GLOW Exosome Labeling Kits (System Biosciences, Palo Alto, California, USA). Five hundred-microliter resuspended exosomes and 50 μl Exo-Red in PBS were incubated at 37 °C for 10 min. The labeling reaction was stopped with 100 μl ExoQuick-TC reagent at 4 °C for 30 min. After centrifugation for 3 min at 14,000 rpm, the labeled exosome pellet was resuspended in 500 μl PBS and monitored under a confocal microscope Leica sp5 with a 63x magnification using the 1.4 oil objective lens (Leica Microsystems, Wetzlar, Germany).

### ELISA

Exosomes were quantified by the Exosome Antibodies & ELISA Kit (System Biosciences), which is specific for the exosomal protein CD63. For performing ELISA, 400 μl plasma was purified from fibrin by adding 4 μl thrombin (BioCat, Heidelberg, Germany) at a final concentration of 5 U/ml. Following exosome extraction by ExoQuick, 50 μl exosome resuspension in duplicate and CD63 protein standards (undiluted, diluted 1:2, 1:4, 1:8, 1:16, 1:32 and 1:64) were added to the micro-titer plate (Tecan). The absorbance at 450 nm of the samples was measured on a spectrophotometric plate reader (Tecan), and the amounts of CD63 protein were calculated according to the exosome protein standard curve.

### Western blot

To calculate the adequate protein amounts for carrying out a Western blot, the protein concentrations were at first measured with the DC Protein Assay Kit (BioRad, Munich, Germany) at a wavelength of 650 nm on a spectrophotometric plate reader (Tecan). A standard curve of 0, 0.625, 1.25, 2.5, 5, and 10 mg/ml BSA (bovine serum albumin; Sigma Aldrich Chemie, Munich, Germany) was applied by the double-dilution method. Five microliters of exosomes, exosome supernatant, and BSA (Sigma Aldrich Chemie) standard protein samples, all solved in RIPA buffer (Merck, Darmstadt, Germany), were added to 96-well plates according to the manufacturer’s instructions. The protein concentrations were then calculated according to a linear equation by applying the regression method.

Exosomes were lysed in RIPA buffer (Merck) and PBS (Life Technologies), and 30 μg of proteins from exosomes and exosome supernatant were electrophoretically separated and blotted onto a PVDF membrane (Millipore, Billerica, USA) which was subsequently incubated with antibodies specific for CD63 (ABGENT, San Diego, California, USA), CD81 (Invitrogen, Darmstadt, Germany) and AGO2 (TAKARA BIO INC, Shiga, Japan) overnight. Detection of the proteins was carried out using peroxidase-conjugated secondary antibodies (Dako, Glostrup, Denmark) and the chemiluminescence ECL detection solution (Sigma-Aldrich, St. Louis, MO, USA).

### Extraction of miRNAs and conversion into cDNA

MiRNAs were extracted from 50 μl exosomes resuspended in 150 μl lysis buffer by using the TaqMan microRNA ABC Purification Kit (Thermo Fisher Scientific, Darmstadt, Germany) according to the manufacturer’s recommendations. The extracted miRNAs were immediately reverse transcribed into cDNA using a modified protocol of TaqMan MicroRNA Reverse Transcription kit (Thermo Fisher Scientific). The 15-μl reaction containing 6 μl Custom RT primer pool, 0.3 μl 100 mM dNTPs with dTTP, 3 μl 50 U/μl MultiScribe Reverse Transcriptase, 1.5 μl 10× RT buffer, 0.19 μl 20 U/μl RNase Inhibitor (Thermo Fisher Scientific), and 4 μl extracted RNA was carried out at 16 °C for 30 min, 42 °C for 30 min, and 85 °C for 5 min on a MJ Research PTC-200 Peltier Thermal Cycler (Global Medical Instrumentation, Ramsey, Minnesota, USA). The cDNA samples were stored at − 20 °C for future use.

### Preamplification of miRNAs

To increase the input cDNA, a preamplification step of cDNA was included. Five-microliter cDNA was preamplified in a 25-μl reaction containing 12.5-μl TaqMan PreAmp Master Mix and 3.75-μl Custom PreAmp Primer Pool (Thermo Fisher Scientific). PCR was run on a MJ Research PTC-200 Peltier Thermal Cycler (Global Medical Instrumentation): 1 cycle at 95 °C for 10 min, 55 °C for 2 min, and 72 °C for 2 min; 16 cycles at 95 °C for 15 s and 60 °C for 4 min; and a terminal cycle 99.9 °C for 10 min. To avoid false-positive data (e.g., primer dimer formation or unspecific PCR products), a negative control without any templates was included from the starting point of all experiments.

### MiRNA expression profiling

To identify differentially expressed miRNAs, real-time TaqMan PCR was at first carried out by using the TaqMan microRNA array Human Pool A cards containing 384 different miRNAs (Thermo Fisher Scientific), and plasma samples from 15 TNBC patients treated with/without chemotherapy and with/without pathological response (pCR). Subsequently, microRNA array cards (Thermo Fisher Scientific) mounted with the 45 most significantly deregulated miRNAs derived from the above array card and 2 endogenous reference miRNAs (miR-92a and miR-484) as well as 1 exogenous control miRNA (cel-miR-39) for data normalization were quantified in plasma of 435 BC patients and 20 healthy women by real-time TaqMan PCR. To carry out real-time TaqMan PCR, the protocol of Thermo Fisher Scientific was modified as followed: The 112.5-μl PCR reaction containing 56.25-μl TaqMan Universal Master Mix II and 2-μl preamplification product was loaded on the array cards. PCR was run on a 7300 HT 384 block (Applied Biosystems): 1 cycle at 95 °C for 10 min and 40 cycles at 95 °C for 15 s, 60 °C for 1 min.

Following 45 miRNAs were selected: snRNU6, let-7g, miR-16, miR-20a, miR-27a, miR-27b, miR-30c, miR-99b, miR-106b, miR-125b, miR-128a, miR-143, miR-145, miR-148a, miR-150, miR-152, miR-155, miR-181a, miR-185, miR-193b, miR-199a-3p, miR-202, miR-301, miR-324-3p, miR-328, miR-335, miR-340, miR-365, miR-370, miR-374, miR-376a, miR-376c, miR-382, miR-410, miR-422a, miR-423-5p, miR-433, miR-489, miR-511, miR-598, miR-628-5p, miR-652, miR-660, miR-744, miR-891a.

As tested by the Genorm Algorithm software, miR-92a and miR-484 provided evidence to be the most suitable reference miRNAs for data normalization.

### Data normalization and statistical analyses

The statistical analyses were performed using the Thermo Fisher Scientific Analysis Software, Relative Quantification Analysis Module, version 3.1 (https://www.thermofisher.com/de/de/home/cloud.html), SPSS software package, version 22.0 (SPSS Inc. Chicago, Illinois, USA) and Statistical Programing Language R, version 3.3.2 (R Core Team 2016, Vienna, Austria).

All raw real-time PCR data were imported into the Thermo Fisher Scientific Analysis Software. First, the amplification curves were manually checked due to their shape of the curve. If a curve was atypical, the Cq value was omitted from the analysis. Second, Cq values with a Cq confidence score below 0.95 were discarded. The Cq confidence score was calculated according to the algorithm implemented in the Thermo Fisher Scientific Analysis Software and describes how likely it is that an obtained Cq value actually comes from a proper amplification curve by assessing the quality of the exponential phase of the respective curve.

The cleaned data were calculated and evaluated by the ΔCq method as follows: ΔCq = value Cq (miRNA of interest) − mean value Cq (reference miR-92a and miR-484). Surprisingly, snRNU6 was not detectable in most plasma samples and could not be used as a normalizer. According to the Genorm Algorithm software, most suitable reference miRNA were miR-92a and miR-484 for data normalization. Thus, normalization of miRNAs of interest was performed with these reference miRNAs [18]. Exogenous cel-miR-39 which was spiked in the plasma samples served as a control for the isolation process.

The Thermo Fisher Scientific Analysis software was used for performing hierarchical clustering (heat map) and Volcano plots. For the heat map, distances between samples and assays were calculated using unsupervised hierarchical clustering based on the ΔCq values and Pearson’s correlation. Clustering method was average linkage. The Volcano plot displays the *p* value versus the fold change for each target in the patient group of interest (BC patient group, HER2-positive or TNBC subgroups) relative to a reference group (healthy group or even HER2-positive subgroup). Here, ΔΔCq was calculated as mean ΔCq (miRNA of interest in the group of interest) − mean of ΔCq (miRNA of interest in the reference group). Then, the relative quantification (Rq or gene expression fold change) was calculated as 2^−(∆∆Cq)^. ΔCq values were used to calculate *p* values using unpaired two-tailed student *t* test, and assuming unequal variances. Subsequently, the relative expression data (Rq) and *p* values adjusted for multiple testing by Benjamini and Hochberg method were log2 and log10-transformed, respectively, and plotted as a volcano plot.

Box plots for the ELISA values of exosomes and data of miRNAs, as well as receiver operating characteristic (ROC) curves, were carried out by the SPSS (version 22) software. For nonparametric comparisons of two dependent and independent variables, miRNA levels before and after therapy and differences in group levels were compared by Wilcoxon and Mann-Whitney *U* tests, respectively.

Fisher’s exact and Mann-Whitney *U* tests were carried out for categorial variables (Table 1) and continuous variables (see Additional file 1: Table S1), respectively. Differences of miRNA levels among the (sub)-groups were calculated using the two-tailed Student *t* test (Table 2). Associations between miRNA levels and dichotomous clinical variables were analyzed by calculating the difference of mean ΔCq values among clinical groups and using an unpaired Student’s *t* test (Table 3). Correlations between miRNA levels and continuous clinical variables are presented by Pearson’s correlation coefficients (see Additional file 1: Table S2). No multiple test correction was applied to the *p* values in Table 3 and Additional file 1: Table S2. The dependency of pCR on miRNA levels, represented by ΔCq values, was estimated from logistic regression models: For each miRNA and each subgroup (all patients, HER2-positive patients, TNBC patients, and patients in the treatment arm), a univariate model as well as a multivariate model including the covariables age (continuous), nodal status (N0 vs. N+), tumor size (T1–2 vs. T3–4), and grading (G1–2 vs. G3) were calculated, and the odd ratio with the 95% confidence interval and the associated Wald *p* value for the miRNA are presented (Table 4). A *p* value < 0.05 was considered as statistically significant. All *p* values are two-sided.

**Table 2:**
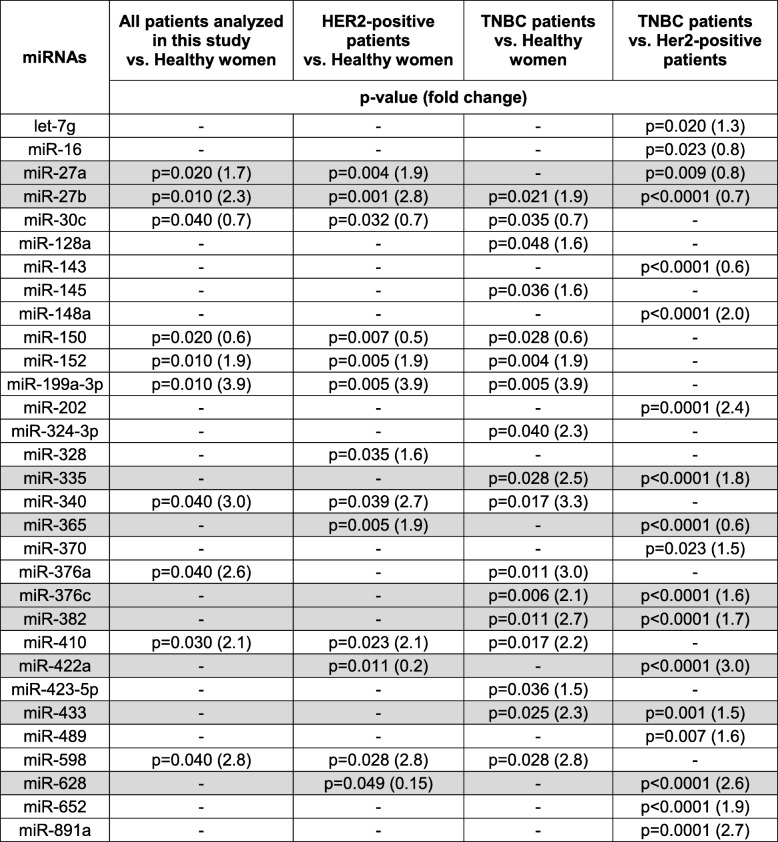
Significantly deregulated exosomal miRNAs in plasma of HER2-positive and TNBC patients

Cells filled with “-” denote insignificant correlations

Exosomal miRNAs levels which are deregulated in one or both subgroups and additionally differ between the two subgroups of HER2-positive and TNBC patients are marked in grey

**Table 3:** Significant associations between the plasma levels of exosomal miRNAs and clinicopathological/risk parameters (categorial variables)

Clinical/risk factors	miRNAs*	All BC patients	HER2-positive patients	TNBC patients
		*p*(*t* test)**	*p*(*t* test)**	*p*(*t* test)**
Age (< 50, ≥ 50)	miR-20a	0.011	–	–
	miR-30c	–	–	0.038
	miR-99b	0.006	–	0.002
	miR-106b	0.024	–	–
	miR-145	0.015	–	0.040
	miR-150	0.008	–	0.015
	miR-185	0.035	–	–
	miR-202	0.046	–	–
	miR-301	0.019	0.032	–
	miR-891a	0.007	–	0.010
Nodal status (N0, N+)	miR-16	–	0.023	–
	miR-328	–	0.019	–
	miR-660	–	0.016	–
Tumor size (T1–2, T3–4)	miR-185	–	0.040	–
	miR-199a-3p	0.034	–	–
	miR-374	–	–	0.030
	miR-376a	–	0.004	–
	miR-382	0.031	0.014	–
	miR-410	–	0.038	–
	miR-433	–	0.037	–
	miR-628-5p	–	0.041	–
Grading (G1–2, G3)	miR-16	0.033	–	–
	miR-20a	0.024	–	0.032
	miR-30c	–	–	0.023
	miR-155	–	–	0.038
	miR-193b	–	–	0.028
	miR-422a	–	0.010	–
	miR-628-5p	–	0.005	–
Lymphocyte predominant breast cancer (neg, pos)	miR-148a	0.036	–	–
	miR-335	0.048	–	–
	miR-652	–	0.040	–
	miR-891a	0.050	0.022	–

Cells filled with “-” denote insignificant correlations

*Only miRNAs are listed which significantly correlate with the clinical parameter in one of the (TNBC and HER2-positive) patient subgroups and/or all patients

**p(*t* test), Student’s *t* test

**Table 4:** Logistic regression models for pCR with *p* values, odds ratio, and confidence intervals

miRNAs	Patients	All BC patients		HER2-positive patients		TNBC patients	
	Model	Univariate	Multivariate	Univariate	Multivariate	Univariate	Multivariate
miR-20a	All	_	_	_	_	_	_
	In PM	_	_	_	_	_	_
	In PMCb	*p* = 0.020 1.39 (1.05–1.84)	*p* = 0.019 1.41 (1.06–1.89)	_	_	_	_
miR-27b	All	_	_	*p* = 0.035 1.30 (1.02–1.65)	*p* = 0.050 1.28 (1.00–1.63)	_	_
	In PM	_	_	_	_	_	_
	In PMCb	*p* = 0.038 1.27 (1.01–1.59)	*p* = 0.030 1.30 (1.03–1.64)	_	_	_	_
miR-99b	All	*p* = 0.103 1.14 (0.97–1.34)	*p* = 0.039 1.19 (1.01–1.41)	_	_	_	_
	In PM	_	_	_	_	_	_
	in PMCb	_	_	_	_	_	_
miR-155	All	*p* = 0.002 1.25 (1.08–1.44)	*p* = 0.003 1.24 (1.08–1.44)	*p* = 0.049 1.24 (1.00–1.53)	*p* = 0.035 1.26 (1.02–1.56)	*p* = 0.013 1.29 (1.05–1.57)	*p* = 0.018 1.29 (1.04–1.58)
	In PM	*p* = 0.033 1.26 (1.02–1.55)	*p* = 0.049 1.24 (1.00–1.53)	_	_	_	_
	In PMCb	*p* = 0.032 1.24 (1.02–1.51)	*p* = 0.023 1.27 (1.03–1.55)	_	_	_	_
miR-193b	All	*p* = 0.039 1.13 (1.01–1.26)	*p* = 0.055 1.12 (1.00–1.26)	*p* = 0.010 1.26 (1.06–1.50)	*p* = 0.012 1.26 (1.05–1.51)	_	_
	In PM	_	_	_	_	_	_
	In PMCb	_	_	_	_	_	_
miR-301	All	*p* = 0.002 1.25 (1.08–1.44)	*p* = 0.001 1.27 (1.10–1.46)	*p* = 0.013 1.30 (1.06–1.60)	*p* = 0.011 1.32 (1.07–1.64)	_	_
	In PM	*p* = 0.040 1.22 (1.01–1.48)	*p* = 0.028 1.25 (1.02–1.52)	_	_	_	_
	In PMCb	*p* = 0.020 1.28 (1.04–1.57)	*p* = 0.022 1.29 (1.04–1.6)	*p* = 0.012 1.53 (1.10–2.12)	*p* = 0.016 1.51 (1.08–2..12)	_	_
miR-365	All	_	_	_	_	_	_
	In PM	_	_	*p* = 0.052 1.35 (1.00–1.81)	*p* = 0.038 1.39 (1.02–1.90)	_	_
	In PMCb	_	_	_	_	_	_
miR-423-5p	All	*p* = 0.048 1.19 (1.00–1.42)	*p* = 0.064 1.18 (0.99–1.41)	_	_	_	_
	In PM	_	_	_	_	_	_
	In PMCb	_	_	_	_	_	_
miR-511	All	_	_	_	_	_	_
	In PM	_	_	_	_	_	_
	In PMCb	_	_	_	_	*p* = 0.239 0.90 (0.76–1.07)	*p* = 0.043 0.78 (0.62–0.99)
miR-628-5p	All	_	_	_	_	*p* = 0.024 0.81 (0.67–0.97)	*p* = 0.017 0.79 (0.65–0.96)
	In PM	_	_	_	_	_	_
	In PMCb	_	_	_	_	_	_
miR-660	All	_	_	*p* = 0.044 1.35 (1.01–1.80)	*p* = 0.027 1.40 (1.04–1.89)	_	_
	In PM	_	_	_	_	_	_
	In PMCb	_	_	_	_	_	_
miR-891a	All	*p* = 0.063 1.07 (1.00–1.15)	*p* = 0.036 1.08 (1.01–1.16)	_	_	_	_
	In PM	_	_	_	_	_	_
	In PMCb	_	_	_	_	_	_

Cells filled with “-” denote insignificant miRNA contributions to the models. MiRNAs which do not show significant contributions in any population were omitted. For each miRNA variable and each patient group, a univariate as well as a multivariate model with the covariables of age, nodal status, tumor size, and grading were calculated

The odds ratio with the 95% confidence interval and the associated Wald *p* value for the miRNAs are presented

*PM* non-carboplatin treatment arm, *PMCb* carboplatin treatment arm

## Results

### Higher levels of exosomes in the blood circulation of BC patients

**Fig. 2:**
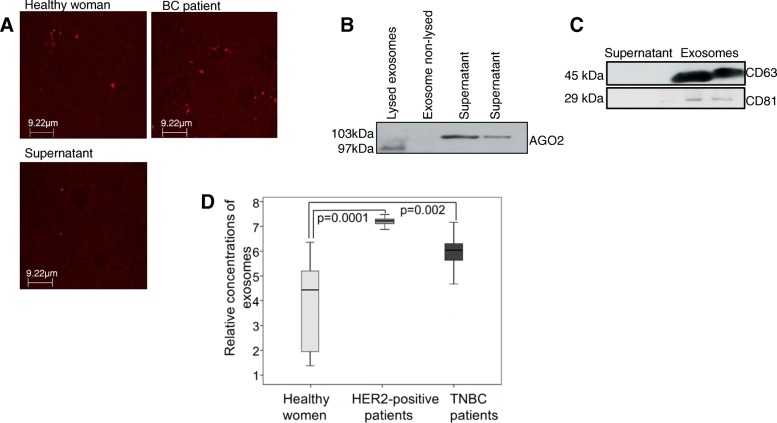
Verification and quantification of exosomes. Exosomes were precipitated from plasma of a healthy woman, a BC patient and supernatant by the agglutinating agent ExoQuick. Exosomes labeled by ExoRed are visible as red dots under the confocal microscope using 63x magnification with a scale bar presented in the picture (**a**). The extraction of exosomes from BC patients was also verified by Western blot using antibodies specific for the exosome proteins CD63 and CD81, and the miRNA-associated AGO2 protein. The Western blot shows a representative example of the supernatant, lysed and non-lysed exosomes where AGO2 protein was detected in lysed exosomes and supernatant (**b**). A further Western blot shows exosomes and supernatant, while in exosomes CD63 and CD81 proteins were identified (**c**)**.** The box plot compares the exosome levels in the plasma of healthy women (*n* = 10), HER2-positive patients (*n* = 78), and TNBC patients (*n* = 40) as measured by an ELISA coated with CD63 antibodies (**d**)

First, we analyzed the exosomes by confocal microscopy, Western blot, and ELISA (Fig. 1). To visualize the exosomes by confocal microscopy, we stained them in plasma of 14 BC patients and healthy women with Exo-Red. As exemplarily shown in the wide-field fluorescence image, the labeled exosomes from a healthy woman, a BC patient, and supernatant are little red dots due to their sizes below the diffraction limit. Some more exosomes can be seen in the plasma of a BC patient than in a healthy woman. In the supernatant, we can also detect a few exosomes, but the level is very low (Fig. 2a). However, it should be kept in mind that these images show only one frame of the pool of exosomes and one time point. The extraction of exosomes from 20 BC patients was also verified on a Western Blot using antibodies specific for the exosomal markers CD63 and CD81, as well as for AGO2. The AGO2-specific antibody recognized cell-free miRNAs bound to AGO2 protein (103-kDa band) in the exosome supernatant, but did not detect AGO2 in the non-lysed exosome pellet. These findings show that the exosome fraction may be pure and devoid of cell-free miRNAs. However, they do not exclude that exosomes may still contain traces of contaminations of cell-free AGO2-bound miRNAs that due to the sensitivity of the Western blot were not detectable. As expected in lysed exosomes, AGO2 protein could be detected; however, its band was at size of around 97 kDa and lower than its bands in the supernatant (Fig. 2b). This discrepancy can possibly be explained due to the fact that supernatant was differently treated and not lysed, and a high concentration of other proteins and contaminants are still available which could produce a shift in size. As described by the company, the AGO2-specific antibody recognizes a band at size of 103 kDa which is detected in the supernatant. Moreover, Sharma et al. showed that the band is at size of 97 kDa corresponding to our findings in the lysed exosomes [19]. However, further analyses have to be carried out to explain this inconsistency. As visible by the 45 and 29 kDa-bands, CD63- and CD81-specific antibodies recognized the non-lysed exosomes in the pellet, respectively, but did not detect any exosomes in the exosome supernatant (Fig. 2c). In contrast to the wide-field fluorescence images that show some exosomes in the supernatant and not in Western blot, these findings indicate that Western blot is not sensitive enough. We also quantified circulating exosomes from plasma of 78 HER2-positive and 40 TNBC patients using an ELISA coated with antibodies against the exosomal marker CD63, and compared their exosome levels with those of 10 healthy women. The exosome levels were significantly higher in HER2-positive (*p* = 0.0001) and TNBC patients (*p* = 0.002) than in healthy women, indicating an excessive, active secretion of exosomes in BC patients. Although the exosome levels were higher in HER2-positive patients than in TNBC patients, the difference between these two levels was not significant (*p* = 0.086, Fig. 2d).

### Different exosomal miRNA signatures in HER2-positive and TNBC patients

Following the qualitative and quantitative analyses of exosomes, we determined the miRNA expression profiles in exosomes derived from plasma of 15 TNBC patients before neoadjuvant therapy using a quantitative TaqMan real-time PCR-based microRNA array card containing 384 different miRNAs (Fig. 1). The patient group included 8 patients treated with carboplatin, (4 with pCR and 4 without pCR), and 7 patients from the non-carboplatin arm (4 with pCR and 3 without pCR). We aimed to select from the panel of 384 miRNAs those exosomal miRNAs which are most differentially expressed between the respective subgroups defined by pCR and treatment arm. While the plasma levels of only one exosomal miRNA (miR-199a, *p* = 0.036) differed between patients with and without pCR, the levels of 4 exosomal miRNAs (miR-125, *p* = 0.029; miR-193b, *p* = 0.029; miR-365, *p* = 0.029; miR-370, *p* = 0.016) differed according to the treatment arm (data not shown). These 5 miRNAs and 40 additional miRNAs that were significantly associated with other clinical parameters (tumor size, nodal status, grading) were selected and mounted (together with two references and one exogenous control miRNA) on 48-microRNA array cards, and further analyzed in plasma from 435 BC patients before treatment and 20 healthy women (Fig. 1). The complete list of miRNAs of this 48-microRNA array card is described in the “Methods” section. The ΔCq values of all 45 miRNAs vs. the mean of references miR-92a and miR-484 among all 455 samples were median-centered and clustered by unsupervised hierarchical clustering based on average linkage and Pearson’s correlation as distance metric. The resulting heatmap shows conspicuously an integrated dark green color of some columns on left side referring to the mean levels of exosomal miRNAs detected in plasma of healthy women suggesting there is no change in their miRNA expression in contrast to the patients. The color scale under the heat map represents ΔCq from the median of all data (see Additional file 1: Figure S2).

**Fig. 3:**
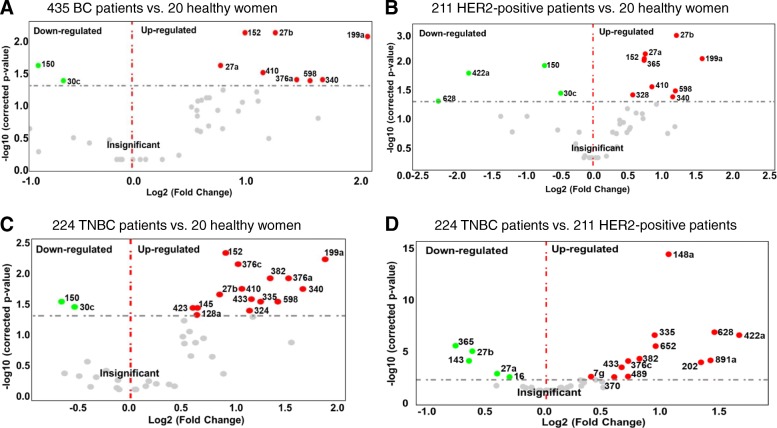
Volcano plot of 45 exosomal miRNAs. Volcano plots of *p* values vs fold changes compare the expression of exosomal miRNAs in 435 BC (**a**), 211 HER2-positive (**b**), and 224 TNBC patients (**c**) with that of 20 healthy women, as well as between HER2-positive and TNBC patients (**d**). The grey dashed line refers to the threshold value corresponding to a corrected *p* value of *p* = 0.05. Significantly downregulated exosomal miRNAs are shown as green dots, significantly upregulated exosomal miRNAs as red dots. Grey dots represent non-significant changes. *p* values are calculated by the Student *t* test and corrected according to the Benjamini and Hochberg method

**Fig. 4:**
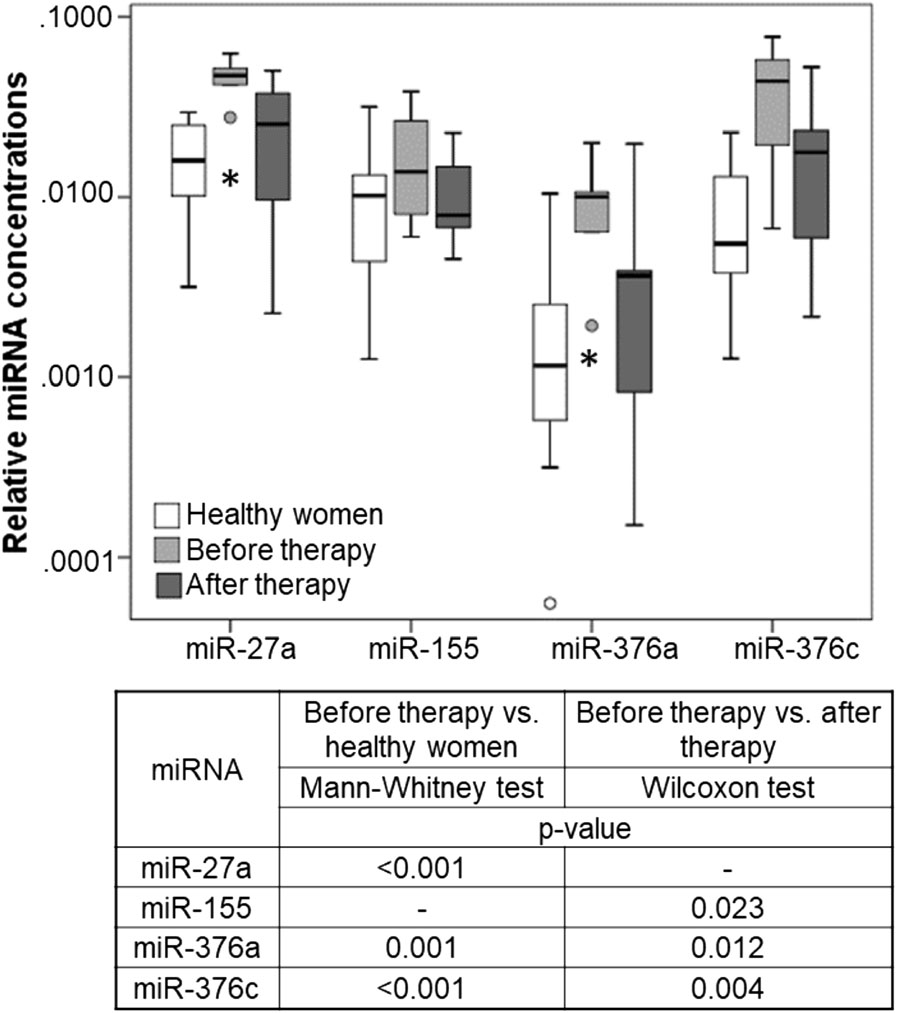
Exosomal miRNA levels before and after neoadjuvant therapy. The box blot shows the plasma levels of exosomal miRNAs of 9 BC patients before and after neoadjuvant therapy and 20 healthy women. *p* values comparing the expression levels before and after therapy, and between patients and healthy women are indicated in the table below the blot. Cells filled with “-” denote insignificant correlations

The volcano plots with the log2 fold changes plotted on the *x*-axis and the negative log10 *p* values plotted on the *y*-axis show all down- (left side) and upregulated (right side) plasma levels of exosomal miRNAs in BC patients. As shown in Fig. 4, the plots compare the expression levels of exosomal miRNA in plasma of all 435 BC patients (A) and the subgroups of 211 HER2-positive BC (B) and 224 TNBC patients (C) with those of 20 healthy women, as well as the levels between TNBC and HER2-positive BC patients (D). Compared with healthy women, we identified 8 up- (red dots) and 2 downregulated (green dots), 9 up- and 4 downregulated and 15 up- and 2 downregulated exosomal miRNAs in the entire cohort of BC patients (A), and in the subgroups of HER2-positive BC (B) and TNBC patients (C), respectively. The levels of 18 exosomal miRNAs differed between TNBC and HER2-positive BC patients, whereby 5 and 13 miRNAs were higher and lower in HER2-positive than in TNBC patients (D), respectively (Fig. 3).

Table 2 summarizes the significant results with the adjusted *p* values and fold changes of miRNAs as derived from volcano plots (Fig. 3). From 45 miRNAs, 30 exosomal miRNAs were either differentially expressed in the subgroups of HER2-positive and TNBC patients, or in all BC patients compared with those of healthy women. Of particular interest are the relative differences of exosomal miRNA levels between HER2-positive and TNBC patients. From the 18 exosomal miRNAs, whose levels differed significantly between HER2-positive and TNBC patient subgroups, 9 miRNAs were also deregulated in one or both subgroups compared with healthy women (Table 2).

With respect to the subgroup, the significant differences between TNBC and HER2-positive patients were reflected by AUC values of 0.737, 0.655, and 0.759 for miR-335, miR-422a, and miR-628, respectively. To improve the discrimination, the concentrations of exosomal miR-335 and miR-628 as well as miR-335, miR-422a, and miR-628 were combined by logistic regression. The combined scores of these exosomal miRNAs could discriminate between TNBC and HER2-positive patients with a sensitivity of 65% and 68% and a specificity of 84% and 81%, respectively (these numbers may be biased towards higher values, because the scores were fitted on the same data). Sensitivities and specificities were determined at the highest Youden index (sensitivity + specificity − 1) (see Additional file 1: Figure S3).

### Exosomal miRNA levels after neoadjuvant therapy

Plasma samples from only 9 BC (4 HER2-positive and 5 TNBC) patients were available directly after neoadjuvant therapy before surgery (Fig. 1). To obtain information on changes in the plasma levels induced by the therapy, we compared the levels of exosomal miRNAs after therapy with those before therapy, and those of healthy women. Only 4 miRNAs (miR-27a, miR-155, miR-376a, and miR-376c) significantly changed their levels after therapy. Since the levels of the other miRNAs hardly differed between before and after therapy, the box plot and the table (*p* values) only show the dynamics of these 4 miRNAs (Fig. 4). Although the data are not representative because of the small number of BC patients, they nevertheless show that the decrease in the levels of 4 exosomal miRNAs to normal (healthy) levels after therapy may be affected by neoadjuvant therapy (Fig. 4). Unfortunately, the cohort of 9 BC patients was too small to result in a robust statistical evaluation.

### Associations of exosomal miRNA levels with the established risk factors

Table 3 (categorial variables) and Additional file 1: Table S2 (continuous variables) summarize the significant correlations between the exosomal miRNA levels and the clinicopathological risk parameters of BC patients. Strikingly, the levels of miRNAs in both subgroups (HER2-positive and TNBC) displayed a different preference to correlate with clinicopathogical parameters: With only one exception (miR-152 and stromal lymphocytes, see Additional file 1: Table S2), no miRNA correlated with a clinical parameter, such as nodal status, tumor size, grading, lymphocyte predominant BC, Ki67 expression, and intratumoral lymphocytes, in both subgroups. In particular, the levels of exosomal miR-16 (*p* = 0.23), miR-328 (*p* = 0.19), and miR-660 (*p* = 0.016) were associated with lymph node status in HER2-positive patients (Table 3). Accordingly, the levels of exosomal miR-16 were lower in TNBC than in HER2-positive patients (*p* = 0.023), while those of miR-328 were only upregulated in HER2-positive patients compared to healthy patients (*p* = 0.035, Table 2). In the subgroup of TNBC patients, only the levels of exosomal miR-374 were associated with higher tumor size, whereas in HER2-positive BC patients, the levels of 6 exosomal miRNAs (miR-185, miR-376a, miR-382, miR-410, miR-433, and miR-628) were associated with the tumor size (Table 3). These findings show the heterogeneity of both BC subtypes that is reflected by the subtype-specific miRNA expression or packaging of miRNAs into exosomes or both, and the relationship of these unique exosomal miRNA patterns with the diverse clinical parameters.

### Associations of exosomal miRNA levels with pCR and treatment arm

Finally, univariate as well as multivariate (with covariables age, nodal status, tumor size and grading) logistic regression models for pCR were carried out in all patients and in the subgroups defined by TNBC patients, HER2-positive patients, and patients in the carboplatin (pMCb) and non-carboplatin (pM) arm. Table 4 contains the unit odds ratio with 95% confidence interval and the corresponding Wald *p* value for the miRNA variable in each model; model results are only reported if the uni- or multivariate model showed a significant contribution of the miRNA to the model, and only miRNAs contributing to all or the single subgroups are reported. At the beginning of our study quantifying exosomal miRNAs in plasma of 8 patients treated with carboplatin (4 with pCR and 4 without pCR), and 7 patients from the non-carboplatin arm (4 with pCR and 3 without pCR) using the microRNA array containing 384 different miRNAs, we detected that the plasma levels of miR-199a (*p* = 0.036) differed between patients with and without pCR, and the levels of miR-125 (*p* = 0.029), miR-193b (*p* = 0.029), miR-365 (*p* = 0.029) and miR-370 (*p* = 0.016) differed according to the treatment arm (data not shown). Now, in uni- and multivariate models comprising our large cohort of 435 patients including the single subgroups, the levels of exosomal miR-199a and the other 4 miRNAs did not correlate with pCR and the treatment arm, respectively, any more, suggesting that our starting patient cohort of 15 patients was too small to establish a significance of these exosomal miRNAs with pCR or treatment arm. In addition, the concentrations of no single miRNA in our set of 45 exosomal miRNAs were associated with the treatment arm, indicating that this set of miRNAs measured in pretreatment plasma samples cannot predict the treatment arm. However, 12 miRNAs could predict pCR in uni- or multivariate models comprising all patients or the single subgroups. Strikingly, the levels of miR-155 most significantly predicted pCR in uni- (*p* = 0.002) and multivariate model (*p* = 0.003) comprising all patients, as well as HER2-positive patients and TNBC patients (Table 4). This exosomal miRNA was also significantly downregulated in the 9 patients after therapy (Fig. 4; *p* = 0.023). Furthermore, the levels of miR-301 were also most significantly associated with pCR in uni- (*p* = 0.002) and multivariate model (*p* = 0.001) comprising all patients, as well as HER2-positive patients. Both the levels of miR-155 and miR-301 correlated somewhat better with pCR in the PMCb than PM arm (Table 4), indicating an improved response to carboplatin-based therapy.

## Discussion

Molecular classification of BC into HER2-positive and TNBC tumors is essential for optimal use of current therapies and for development of new drugs. Of interest is that exosomes participate in cell-to-cell communication between cancer cells and normal host cells, and thus, are crucial components for regulation of the tumor microenvironment [20]. In this regard, investigation of the involvement of exosomal miRNAs of these tumors could provide new diagnostic/prognostic biomarkers and therapeutic target molecules, apart from a better understanding of tumor growth processes. In the current study, we identified miRNA signatures in exosomes specific to discriminate between HER2-positive and TNBC patients, indicating the different biology in these subgroups. Strikingly, different exosomal miRNA patterns were associated with the clinicopathological characteristics within the respective subgroups. As far as we know, this is the first study that measured a panel of 45 miRNAs in exosomes derived from a large cohort of 435 BC patients.

As we recently reported for ovarian cancer patients [21], we found that BC patients also had an excessive, active secretion of exosomes into their blood circulation. Although the levels of exosomes were somewhat higher in HER2-positive than TNBC patients, the difference was not significant. These findings suggest that a high secretion of exosomes may be a general feature of cancer patients. However, the exosomes differed in their content within both subgroups. Namely, we detected differently expressed miR-27a/b, miR-335, miR-365, miR-376c, miR-382, miR-422a, miR-433, and miR-628 in exosomes of either HER2-positive or TNBC patients compared with healthy women. This subtype-specific distribution of miRNAs in exosomes may indicate both, a different miRNA expression pattern and a selective exosomal packaging process. Based on the ability of exosomes to communicate between cells, the detection of these miRNA panels in exosomes may be superior to the detection of cell-free miRNAs in plasma or serum. To date, the presence of these miRNAs has not yet been described in BC-derived exosomes.

In our study, we detected that in comparison with healthy women, the levels of exosomal miR-27a were only significantly upregulated in HER2-positive patients (but not in TNBC patients), whereas the levels of miR-27b were upregulated in both subtypes, but with a significantly higher exosomal occurrence in HER2-positive patients. MiR-27a was reported to activate the Wnt/β-catenin signaling pathway to promote the proliferation, migration, and invasion of BC cells [22]. So far, an association of miR-27a with HER2-positive BC has not been described. However, in contrast to our data an association of this miRNA with TNBC was revealed in a meta-analysis, but its quantification was carried out in tumor tissues and not in exosomes [23]. These findings possibly point to a selective packaging of miRNAs in exosomes independent of their expression levels. Conversely, our findings on the higher levels of exosomal miR-27b in HER2-positive patients are supported and complemented by the data by Jin et al. [24] showing that HER2 stimulated miR-27b expression through the AKT/NF-κB signaling cascade. We also detected that the levels of exosomal miR-27b predict pCR in HER2-positive patients, indicating its narrow association with HER2-positive tumors. Despite the conventional role of miR-335 to act as a tumor suppressor in BC [25], our data demonstrate its significantly increased occurrence in exosomes from TNBC patients. Nevertheless, Martin et al. [26] reported that miR-335 may also act in an oncogenic way in BC, to repress genes involved in the ERα signaling pathway, and consequently, to enhance resistance to the growth inhibitory effects of tamoxifen. Contrary to our findings that show significantly upregulated levels of exosomal miR-365 in the subgroup of HER2-positive (but not in TNBC), miR-365 was reported to be downregulated and act as a tumor suppressor in BC. Kodahl et al. [27] showed that its expression levels were lower in serum of ER-positive BC patients than healthy controls, whereas we show that its levels in HER2-positive patients who do not express ER were increased. In addition, miR-365 was also described to be oncogenic. Overexpression of miR-365 promoted cell proliferation and invasion through targeting ADAMTS-1 (a disintegrin and metalloproteinase with thrombospondin motifs) in BC cells [28]. In our study, significantly higher levels of exosomal miR-376c and miR-382 were observed in TNBC patients, but not in HER2-positive BC patients. Upregulated levels of miR-376c [29] and miR-382 [30] were also detected in plasma and serum of BC patients (regardless of the subtypes), respectively, by two previous studies. In BC, miR-382 targeted and repressed the Ras GTPase superfamily member RERG (Ras-related and estrogen-regulated growth inhibitor), to attenuate the inhibitory effects of RERG on the oncogenic Ras/ERK pathway. Thereby, miR-382 promoted BC cell viability, clonogenicity, survival, migration, invasion and in vivo tumorigenesis/metastasis [31]. Contrary, for example in oral squamous cancer, miR-376c seems to have tumor suppressive functions. Its overexpression in these cancer cells suppressed fission, proliferation, migration and invasion and induced cell apoptosis via targeting the transcription factor HOXB7 [32]. Finally, we found that the levels of exosomal miR-422a were downregulated in HER2-positive BC patients, whereas the levels of exosomal miR-433 were upregulated in TNBC patients, but till now, quantitative data on these miRNAs have not been published for BC patients. It was reported that in BC stem cells, upregulation of miR-422a attenuated microsphere formation, proliferation, and tumor formation via suppressing the PLP2 (Proteolipid protein 2) expression [33]. Moreover, miR-433 repressed Rap1a, a small G protein of the Ras guanosine triphosphatase (GTPase) superfamily that activates the MAPK signaling pathway, and thus repressed cell migration and proliferation and induced apoptosis in BC [34]. In addition, miR-433 targeted AKT3 in BC [35]. These findings highlight miR-422a and miR-433 as tumor suppressor genes.

Not only the miRNA patterns in exosomes differed between HER2-positive and TNBC patients, but they were also specifically associated with different clinicopathological parameters within the subgroups. For example, we identified a particular set of exosomal miRNAs (miR-16, miR-328, and miR-660) to be associated with lymph node status only in the subgroup of HER2-positive BC patients, but not in TNBC patients. In addition, we detected that miR-660 predicted pCR to neoadjuvant therapy in HER2-positive patients. Shen et al. already showed the potential of miR-660 as a therapeutic target for clinical treatment of BC, and its role as a regulator of proliferation, migration, and invasion of human BC cells [36]. Moreover, our present findings on the association of the levels of exosomal miR-16 with lymph node status are substantiated by our previous data [37], demonstrating such an association with cell-free miR-16 in plasma. Thus, our findings indicate a possible role of miR-16 in the development of lymph node metastases in BC. In the subgroup of TNBC patients, we discovered that only the levels of exosomal miR-374 were associated with a higher tumor size, whereas the levels of 6 exosomal miRNAs (miR-185, miR-376a, miR-382, miR-410, miR-433, and miR-628) showed such an association in HER2-positive BC patients. In addition, we revealed that miR-376a, those exosome-free plasma levels, are also upregulated in BC [29] displayed a dynamic presence in exosomes. Aside from miR-376a, three further miRNAs (miR-27a, miR-155, and miR-376c) were also downregulated to normal levels after neoadjuvant therapy, suggesting that these miRNAs may be released from the primary tumor into the blood to some extent, and their changes may directly reflect cancer status. Especially, miR-155 is a well-known miRNA with both tumor suppressive and oncogenic character, targeting, e.g., HER2 [38] and the transcription factor FOXO3a [39, 40] in BC, respectively. Along with miR-27a, there is also an association of miR-155 with the decreased expression of FOXO3a which is paralleled with the increased expression of RUNX2 [41].

In neoadjuvant settings, the early identification of non-responding BC is crucial to avoid ineffective treatments. In particular for aggressive TNBC and HER2-positive BC subtypes, achievement of pCR correlates with improved long-term outcome [16, 42]. Here, we show for the first time that the levels of exosomal miR-155 in all BC patients and their subgroups, as well as exosomal miR-301 with the exception of triple-negative BC patients most significantly predicted pCR to neoadjuvant therapy. This information could be used for treatment stratification considering alternative treatment options. However, to introduce exosomal miR-155 and miR-301 as predictive markers, further prospective studies are necessary to confirm their predictive value. Particularly, the quantification of these exosomal miRNAs in a large cohort of plasma samples before, during, and after chemotherapy is required. Since miR-301 regulates the PTEN/Akt and NFκB signaling pathways that are important in the progression of BC [43, 44], and binds to estrogen receptor 1 mRNA leading to estrogen independence of BC [45], miR-301 may be an early therapeutic target molecule in BC.

To summarize, our findings suggest that certain miRNAs are selectively enriched in exosomes of HER2-positive and TNBC patients and are also associated with the clinicopathological parameters and pCR within the BC subtypes. Exosomal miRNAs may reflect the characteristics of their parental cells and, therefore, may offer a tumor-related profile. Recently, we found that the majority of miRNAs detectable in plasma is concentrated in exosomes [2]. However, it is of note to mention that the plasma exosome population is a heterogeneous mixture of cancer and normal (wild type) exosomes, and may be derived from all cells types, especially from blood cells. This, of course, compromises the tumor specificity of the identified exosomal miRNA signatures. Therefore, methods to selectively enrich cancer exosomes from plasma or serum have to be advanced. Unfortunately, tumor-associated exosome markers allowing such an enrichment are poorly defined. In addition, our unpublished data show that the proportion of tumor-derived exosomes is small in comparison with normal, wild-type exosomes impeding the isolation of low-abundant miRNAs. However, the extensive secretion of exosomes in BC patients triggered by the tumor points to that the tumor also communicates with wild-type exosomes. Thus, we should keep in mind that not only exosomes derived from the primary tumor or metastases may be eligible for cancer personalized diagnostics, but also exosomes derived from other organs that are affected by tumor burden [46].

Although our results show a different packaging of miRNAs into exosomes, and exosomal miRNAs as future diagnostic markers and therapeutic molecules, there are some aspects that may limit our study. Our analyses were carried out by miRNA array cards. We did not verify them by single real-time PCR assays, since the number of miRNAs was too high, and the population size too large in our study. However, our previous analyses in an independent cohort before starting the current study showed that the data were nearly congruent applying miRNA array cards and single real-time PCR analyses. In addition, the number of plasma samples collected directly after neoadjuvant therapy was too low, to make a statistical conclusion on the impact of therapy on the miRNA expression levels. However, the strength of our study is the number of miRNAs analyzed and the size of our patient population before neoadjuvant therapy.

## Conclusion

Our data demonstrate differentially expressed and packaged miRNA sets in BC exosomes that could serve as potentially diagnostic and therapeutic markers for BC. These specific exosomal miRNA profiles that exclusively reflect HER2-positive and TNBC as well as the different stages of BC may provide insight into the exosome biology for monitoring the disease. Further analyses are planned to analyze the significantly deregulated exosomal miRNAs in a higher number of plasma samples collected after treatment as well as in follow-up studies. Finally, a detailed investigation on their association with pCR and treatment arms is required.

## Additional file

Additional file 1: **Figure S1.** Levels of free hemoglobin were measured in plasma samples by spectrophotometry at wavelengths from 350 to 650 nm. A dilution series of lysed red blood cells in plasma was prepared (below the chart). The degree of hemolysis was determined based on the optical density (OD) at 414 nm (absorbance peak of free hemoglobin, called Soret band), with additional peaks at 541 and 576 nm. Samples were classified as being hemolysed if the OD at 414 exceeded 0.25. The integrated curve of BC plasma samples comprises values from 0.08 to 0.20 indicating that the samples were non-hemolysed. **Figure S2.** Hierarchical cluster is shown by heat map of median centered ΔCq values of exosomal miRNAs (in rows) derived from plasma samples of 435 BC patients before treatment and 20 healthy women (in columns). The red and green colors indicate that the ΔCq values are below (relatively high expression) and above (relatively low expression levels) the median of all ΔCq values in the study, respectively. Bottom: clustering of samples. Left: clustering of probes. The scale bar provides information on the degree of regulation. The 5 clinically relevant miRNAs derived from the microRNA array cards containing 384 different miRNAs are indicated by a red arrow. **Figure S3.** Exosomal miRNAs differ between HER2-positive and TNBC patients. ROC analyses show the profiles of sensitivity and specificity of exosomal miR-335, miR-422a, and miR-628 and their combinations to distinguish TNBC from HER2-positive BC patients. The table below the ROC shows the summarization of sensitivities and specificities of exosomal miR-335, miR-422a, miR-628, and their combinations. **Table S1.** Patient characteristics at the time of primary diagnosis of breast cancer (continuous variables). **Table S2.** Significant associations between the plasma levels of exosomal miRNAs and clinicopathological risk parameters (continuous variables). (ZIP 2140 kb)

## Abbreviations

3′UTR: 3′ Untranslated-region
ADAMTS-1: A disintegrin and metalloproteinase with thrombospondin motifs
AUC: Area under the curve
BC: Breast cancer
ER: Estrogen receptor
HER2: Human epidermal growth factor receptor 2
miRNA: MicroRNA
pCR: Pathological complete response
pM: Non-carboplatin arm
pMCb: Carboplatin arm
PR: Progesterone receptor
RERG: Ras-related and estrogen-regulated growth inhibitor
ROC: Receiver operating characteristic
TNBC: Triple-negative breast cancer
