# Supplementary material for: Specific microRNA signatures in exosomes of triple-negative and HER2-positive breast cancer patients undergoing neoadjuvant therapy within the GeparSixto trial
Source: BMC Med. 2018 Oct 10;16:179. doi: 10.1186/s12916-018-1163-y (PMC6178264; doi:10.1186/s12916-018-1163-y)
Supplement: Supplementary file 1 — Figure S1. Levels of free hemoglobin were measured in plasma samples by spectrophotometry at wavelengths from 350 to 650 nm. A dilution series of lysed red blood cells in plasma was prepared (below the chart). The degree of hemolysis was determined based on the optical density (OD) at 414 nm (absorbance peak of free hemoglobin, called Soret band), with additional peaks at 541 and 576 nm. Samples were classified as being hemolysed if the OD at 414 exceeded 0.25. The integrated curve of BC plasma samples comprises values from 0.08 to 0.20 indicating that the samples were non-hemolysed. Figure S2. Hierarchical cluster is shown by heat map of median centered ΔCq values of exosomal miRNAs (in rows) derived from plasma samples of 435 BC patients before treatment and 20 healthy women (in columns). The red and green colors indicate that the ΔCq values are below (relatively high expression) and above (relatively low expression levels) the median of all ΔCq values in the study, respectively. Bottom: clustering of samples. Left: clustering of probes. The scale bar provides information on the degree of regulation. The 5 clinically relevant miRNAs derived from the microRNA array cards containing 384 different miRNAs are indicated by a red arrow. Figure S3. Exosomal miRNAs differ between HER2-positive and TNBC patients. ROC analyses show the profiles of sensitivity and specificity of exosomal miR-335, miR-422a, and miR-628 and their combinations to distinguish TNBC from HER2-positive BC patients. The table below the ROC shows the summarization of sensitivities and specificities of exosomal miR-335, miR-422a, miR-628, and their combinations. Table S1. Patient characteristics at the time of primary diagnosis of breast cancer (continuous variables). Table S2. Significant associations between the plasma levels of exosomal miRNAs and clinicopathological risk parameters (continuous variables). (ZIP 2140 kb) [file 12916_2018_1163_MOESM1_ESM.zip › Table S1R2.docx]

**Table S1 Patient characteristics at the time of primary diagnosis of breast cancer (continuous variables)**

| **Parameters** | | **All BC patients analyzed in this study** | **HER2-positive patients** | **TNBC patients** | **All BC patients**  **in GeparSixto trial** | **p-value*** |
| --- | --- | --- | --- | --- | --- | --- |
| Ki67*** | mean | 46 | 32 | 59 | 47 | 0.4179 |
|  | std** | 25 | 18 | 23 | 25 |  |
|  | median | 40 | 30 | 60 | 40 |  |
|  | min, max | 3, 95 | 3, 90 | 8, 95 | 2, 95 |  |
| Stromal lymphocytes, (strLy)*** | mean | 32 | 30 | 35 | 32 | 0.4678 |
|  | std** | 25 | 25 | 24 | 25 |  |
|  | median | 30 | 20 | 30 | 30 |  |
|  | min, max | 0, 95 | 0, 95 | 0, 90 | 0, 95 |  |
|  | missing | 5 | 5 | 0 | 7 |  |
| Intratumoral lymphocytes, (iTuly )*** | mean | 9 | 6 | 12 | 9 | 0.5981 |
|  | std** | 12 | 8 | 14 | 12 |  |
|  | median | 5 | 5 | 10 | 5 |  |
|  | min, max | 0, 70 | 0, 60 | 0, 70 | 0, 70 |  |
|  | missing | 7 | 7 | 0 | 9 |  |
| Body mass index | mean | 25.3 | 25.3 | 25.3 | 25.4 | 0.9702 |
|  | std** | 4.6 | 4.6 | 4.6 | 4.8 |  |
|  | median | 24.4 | 24.8 | 24.3 | 24.5 |  |
|  | min, max | 17.9, 47.4 | 17.9, 47.4 | 18.2, 42.2 | 16.7, 47.4 |  |

*Characteristics of patients were compared between patients with analyzed samples and all patients of GeparSixto study (modified intend-to-treat population using Wilcoxon tests.

**std, standard deviation.

***measured % at baseline.
